# Carcinome basocellulaire térébrant

**DOI:** 10.11604/pamj.2018.30.300.16224

**Published:** 2018-08-30

**Authors:** Jawad El-Azhari, Mohammed Boui

**Affiliations:** 1 Service de Dermatologie-Vénérologie, Hôpital d’Instruction Mohammed V, Rabat, Maroc

**Keywords:** Carcinome basocellulaire térébrant, malignité locale, bilan d´extension, Terebrant basal cell carcinoma, local malignancy, staging evaluation

## Abstract

Basal cell carcinoma (BCC) is the most frequent skin cancer. It never affects the mucosa. It is characterized by a local malignancy with an extremely rare risk of developing distant metastasis and the lowest mortality. However in the absence of diagnosis and early surgery, BCC can be locally invasive and result in severe tissue destruction. It can ulcerate and its growth can be extensive and destructive: terebrant tumors affecting the structures of the muscles and the bone. We here report the case of a 80-year old woman living in an isolated rural areas, presenting with ulcerated exudative and smelly burgeoning lesion in the pretragal and the parotid regions. Locoregional examination was unremarkable and histology showed infiltrative BCC. Staging evaluation objectified no bone invasion. The patient was referred to the Division of Plastic Surgery for carcinologic treatment.

## Image en médecine

Le carcinome basocellulaire (CBC) est le plus fréquent des cancers avec une localisation strictement cutanée et jamais muqueuse. Il possède une malignité locale et son risque métastatique est exceptionnel. Il possède la mortalité la plus faible. Cependant en l'absence de diagnostic et chirurgie précoces, le CBC a un potentiel invasif local qui peut entraîner une destruction tissulaire importante. Le CBC peut s'ulcérer et avoir une évolution extensive et destructrice: on parle de formes térébrantes pouvant atteindre les structures musculaires et osseuses. Nous rapportons le cas d'une femme de 80 ans, habitant en zone rurale enclavée, consulte pour une lésion ulcéro-bourgeonnant; prenant les régions prétragienne et parotidienne; exsudative et nauséabonde. L'examen locorégional était sans particularités et l'histologie était en faveur d'un CBC infiltrant. Le bilan d'extension n'a pas montré d'envahissement osseux. La patiente a été adressée au service de chirurgie plastique pour une prise en charge carcinologique.

**Figure 1:**
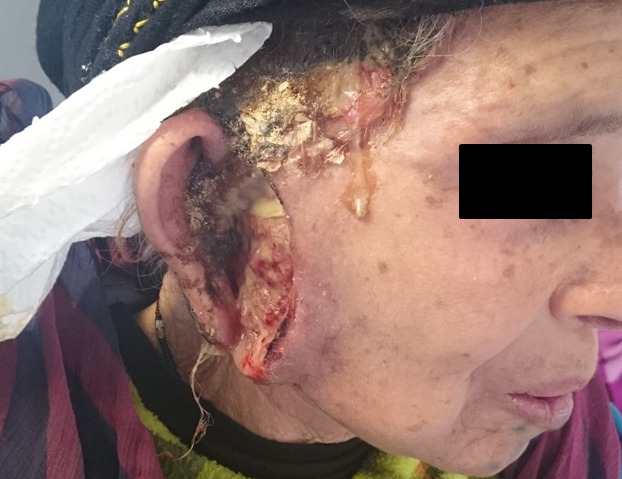
Lésion ulcéro-bourgeonnant exsudative des régions prétragienne et parotidienne

